# Investigating the Locomotion of the Sandfish in Desert Sand Using NMR-Imaging

**DOI:** 10.1371/journal.pone.0003309

**Published:** 2008-10-01

**Authors:** Werner Baumgartner, Florian Fidler, Agnes Weth, Martin Habbecke, Peter Jakob, Christoph Butenweg, Wolfgang Böhme

**Affiliations:** 1 Department of Cellular Neurobionics, RWTH-Aachen University, Aachen, Germany; 2 Department of Experimental Physics 5, University of Würzburg, Würzburg, Germany; 3 Institute of Computergraphics and Multimedia, RWTH-Aachen University, Aachen, Germany; 4 Chair of Structural Statics and Dynamics, RWTH-Aachen University, Aachen, Germany; 5 Zoologisches Forschungsmuseum Alexander Koenig (ZFMK), Bonn, Germany; University of Bristol, United Kingdom

## Abstract

The sandfish (*Scincus scincus*) is a lizard having the remarkable ability to move through desert sand for significant distances. It is well adapted to living in loose sand by virtue of a combination of morphological and behavioural specializations. We investigated the bodyform of the sandfish using 3D-laserscanning and explored its locomotion in loose desert sand using fast nuclear magnetic resonance (NMR) imaging. The sandfish exhibits an in-plane meandering motion with a frequency of about 3 Hz and an amplitude of about half its body length accompanied by swimming-like (or trotting) movements of its limbs. No torsion of the body was observed, a movement required for a digging-behaviour. Simple calculations based on the Janssen model for granular material related to our findings on bodyform and locomotor behaviour render a local decompaction of the sand surrounding the moving sandfish very likely. Thus the sand locally behaves as a viscous fluid and not as a solid material. In this fluidised sand the sandfish is able to “swim” using its limbs.

## Introduction

The scincid lizard genus *Scincus* [1] is distributed over an extensive desert belt ranging from the African west coast (Morocco to Senegal) through the Sahara and the Arabian peninsula into Jordan, Iraq and SW Iran [2]–[6]. Within this vast range, which biogeographically resides in Saharo-Sindian type, there are several species of *Scincus scincus* (including *S. s. conirostris*, *S. s. cucullatus*, *S. s. meccensis*), *S. albifasciatus* (including *S. a. laterimaculatus*), *S. hemprichii*, and *S. mitranus*. The two last taxa include other previously recognized species in synonymy [3]. All of these only rarely coexisting forms exhibit the same general behavioural features in being able to not only dive into loose, aeolian desert sand in order to escape from predators (sand diving), but to also move beneath the dry sand surface with considerable speed over significant distances (sand swimming). Niche segregation in sympatric forms (e.g. *Scincus s. conirostris* and *S. mitranus* in Saudi Arabia) is not yet well understood and may relate to the grain size of the sandy substrate. The morphological adaptations related to this lifestyle have long been known [1], [7], [8]. These adaptations include a shovel-shaped snout with the lower jaw wedged beneath the upper jaw, reduced ear openings, a subquadrangular cross section of the body (more precisely, a flattened pentagon), and strongly developed limbs with fringed digits and toes. Recent authors have studied and/or discussed anatomical, morphological and behavioural aspects of the genus *Scincus* (the “sandfish” of the Arabs) in the Saharo-Arabian region [3], [9]–[18].

Movement in or under sand is also used by several other lizards and has been independently acquired in several families (see Table 1). Some of these families only perform sand burying behaviour(sand diving) to hide from predators, especially during night (e.g. some *Liolaemus* species [19]). Others actually move under the sand (sand swimming). The majority of these lizards are characterized by reduced limbs that are adpressed to the body during sand swimming or even by absent limbs (Table 1). These forms move through soil using a serpentine action, and only a few species (see Table 1) with well-developed limbs perform true sand swimming. Whether or not the exact mechanisms of sand swimming are equal to that of *Scincus* is unclear.

**Table 1:** Summary of relevant sand-dwelling squamate reptiles [9], [28].

Family	4 well-developed limbs		limbs reduced or absent
	Sand-diving (burial only)	Sand-swimming (locomotion below sand)	Sand-diving or -swimming
Phrynosomatidae	*Callisaurus draconoides*		
	*Cophosaurus texanus*		
	*Phrynosoma* spp.		
	*Uma notata*		
Tropiduridae	*Liolaemus* spp.		
	*Tropidurus* spp.		
Agamidae	*Agama etoshae*		
	*Phrynocephalus* spp.		
Lacertidae	*Eremias* spp.		
	*Meroles* spp.	*Meroles anchietae*	
Gerrhosauridae	*Angolosaurus skoogi*	*Angolosaurus skoogi*	
Scincidae	*Ctenotus* spp.		*Acontias* spp.
	*Eremiascincus* spp.	*Eremiascincus* spp.	*Chalcides* (*Sphenops*) spp.
	*Scincopus fasciatus*		*Cryptoscincus minimus*
	*Scincus* spp.	*Scincus* spp.	*Lerista* spp.
	*Trachylepis acutilabris*		*Neoseps reynoldsi*
			*Pygomeles* spp.
			
Gymnophthalmidae			*Calyptommatus* spp.
			*Notobachia* spp.
			*Procellosaurinus* spp.
			*Vanzoia rubricauda*
Amphisbaenidae			*Amphisbaena* spp.
			*Bipes* spp.
Trogonophidae			*Agamodon* spp.
			*Diplometopon zarudnyi*
			*Pachypalaminus* sp.
			*Trogonophis wiegmanni*
Leptotyphlopidae			*Leptotyphlops* spp.
Typhlopidae			*Typhlops* spp.
Boidae			*Eryx* spp.
			*Gongylophis* spp.
			*Lichanura* spp.
Colubridae			*Chilomeniscus* spp.
			*Chionactis* spp.
			*Phimophis* spp.
Elapidae			*Simoselaps bertholdi*
Viperidae			*Bitis peringueyi*
			*Cerastes vipera*

The sand-diving techniques of species with well-developed limbs show much variation and have recently been described and discussed in detail [9]. They range from a vertical “burial” of the body by rapid lateral oscillations, as in *Phrynocephalus* and *Phrynosoma*, to the so-called “shimmy” burial of *Uma*, *Callisaurus* and *Cophosaurus*. True sand swimming, however, where a lizard with well-developed limbs remains for longer periods below the surface and covers considerable distances with remarkable speed appears to be restricted to the species of *Scincus* and possibly to the lizard said to be most similar in its sand-swimming behaviour, the gerrhosaurid *Angolosaurus*. It is tempting to assume that the morphologically different lizards *Meroles* (Lacertidae) and *Eremiascincus* (Scincidae) may use a different mode of underground locomotion. It is generally believed that in this locomotory-type propulsive thrust is produced without the use of limbs [11], [17]. Arnold [9] explicitly states that “the forelegs are then laid back along the body as they become submerged but this happens to the hindlimbs, while they are still fully exposed. From this stage, locomotion is essentially serpentine with the body and tail following the high-amplitude sinusoidal curve adopted by the head.” For *Angolosaurus*, sand diving has been described [9] and is stated to be very similar to that of *Scincus* [9], the lizards “moving sinusoidally beneath the sand”.

In this paper, we test this hypothesis of a serpentiform sand swimming with adpressed limbs by *S. scincus* using NMR imaging, because, along with the sensory system, the integument [10] and the breathing mechanism, locomotion seems to be a particularly important adaptation of this desert lizard.

## Results

**Figure 1:**
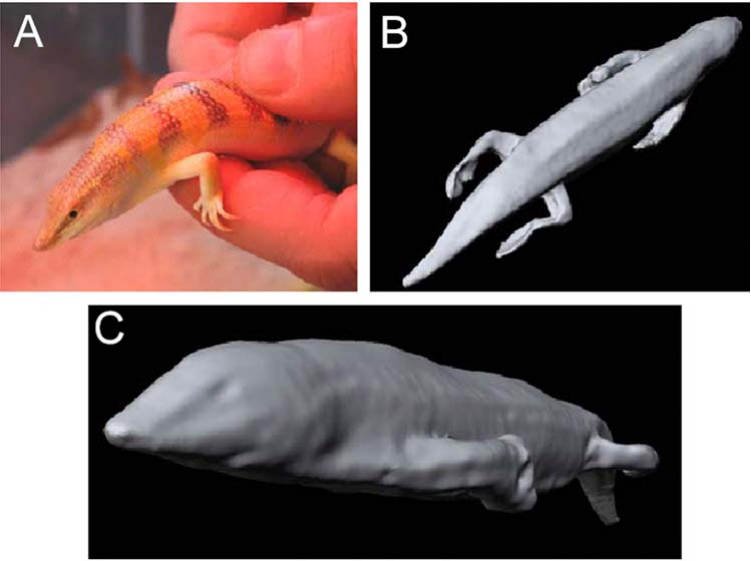
Morphology of a sandfish. (A) A living adult sandfish in the hand of the experimenter. (B) top-view (C) side-view of a 3D-reconstruction of a fixed sandfish. The spatula-shaped snout, the streamlined body shape, the smooth integument, long limbs as well as long and fringed digits can be seen representing typical adaptations to live in lose sand. The transversal section of the skink represents approximately a flattened pentagon pointing dorsal. However, axillary grooves for the limbs are not evident.

Along with conventional photography, 3D-laserscanning was employed to document the macroscopic morphology of *S. scincus*. Typical results are depicted in Figure 1, and the full 3D-model is available in the supplementary data (Figure S1). Typical adaptations to the life in loose sand are evident in the spatula-shaped snout, the streamlined body shape, the smooth integument, and broadened and fringed digits [20]. It can be clearly seen that the limbs are well developed and show no sign of reduction. The transverse section of the skink approximates a flattened pentagon with the apex, pointing dorsal. It has a maximal width of about 20 mm. No axillary grooves for the limbs are evident.

**Figure 2:**
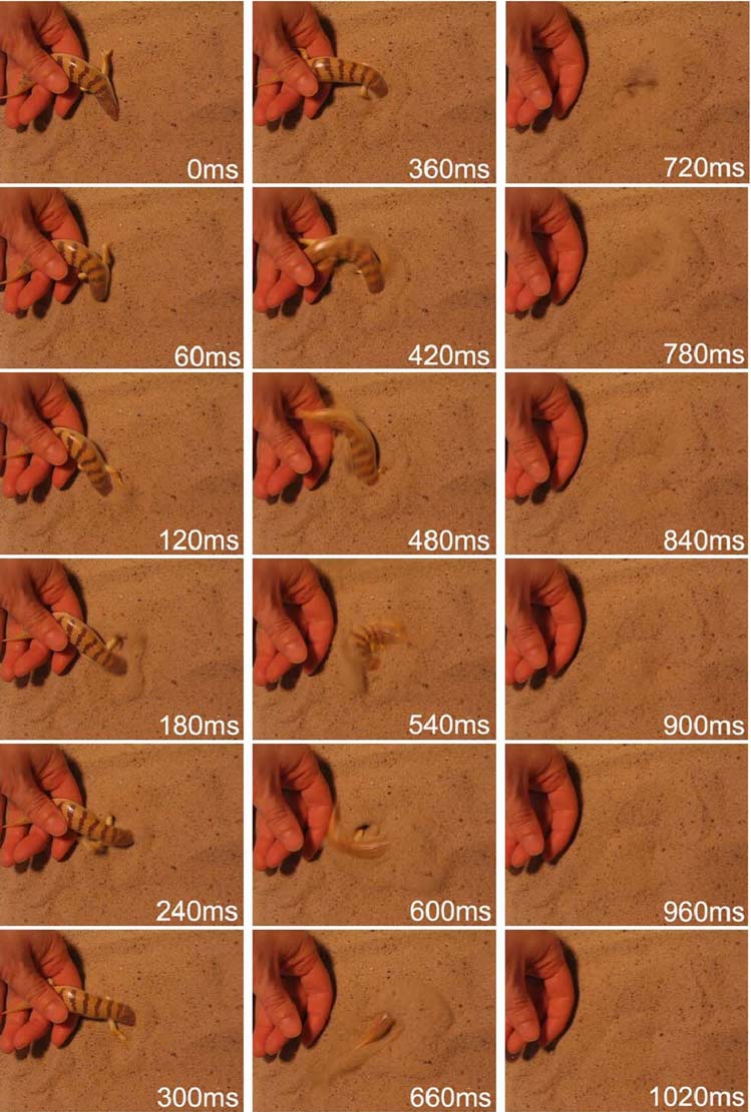
Time series of snapshots of the burying behaviour of the sandfish. After released from the experimenter's hand, the lizard starts to perform a serpentiform movement which is accompanied by a limb movement typically a way that a front limb is “swung” backwards when the cranial part of the lizard is bent towards the colateral side.

The rapid burying process of an adult sandfish released from the experimenter's hand is documented in Figure 2 in 60 ms intervals, and also in a supplementary video (Video S1). The sinuous movement of the body and tail can be clearly seen. Each point on the body undergoes a sinusoidal lateral displacement with amplitudes of about half the body length and a frequency of approximately 3 Hz. Furthermore, it is clearly evident that the limbs are not folded against the body and kept immobile, but are actively used to generate thrust by an alternating paddling-like movement. In all of the videotape sequences of burying sandfish there was no clear indication of additional torsional movements of the body.

**Figure 3:**
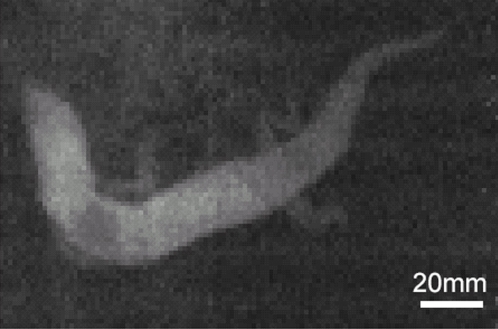
NMR image of a sandfish buried approximately 100 mm deep in loose sand (top-view). - Clearly the body is contorted in a serpentiform manner and the limbs are not laid back along the body as suggested in the literature. The NMR-image is of the same individual shown in Figure 1.

To observe the sandfish in the sand, NMR-imaging, adjusted to detect hydrogen atoms, was employed. This was found to be the best method as the dry sand does not yield a hydrogen NMR-signal whereas the tissues of the sandfish give a clear signal yielding a sufficient signal to noise ratio. This enables fast, repetitive imaging which is necessary for the investigation of locomotion within the sand. An image of a sandfish resting beneath about 100 mm of sand is depicted in Figure 3. The body is flexed in the plane of the image. The limbs are clearly visible and are obviously extended from the body.

**Figure 4:**
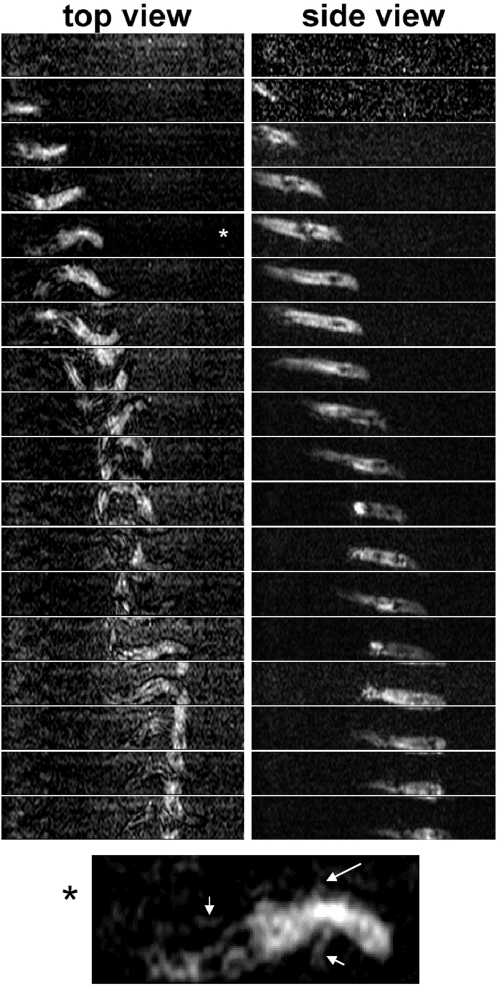
Time series (side- and top-view) NMR-images during the burying process of a sandfish. Whereas a clear serpentiform (sinusoidal) movement can be observed in the top view, no significant contortion of the body can be seen in the side view. Although the SNR is not sufficient to see fine details of the lizard, in several images the limbs can be clearly identified and can be seen to perform a swimming or walking like movement. The time interval tΔ images was set to 120 ms for this experiment. Due to the NMR-protocol used, the object leaving the imaging area at the top side appears to enter at the bottom side and vice versa as explained in detail in the Methods. The image marked with asterisk is shown at the bottom in detail showing three visible limbs extended from the body (legs marked by arrows).

Using the projection method described in the “Materials and Methods” section, we were able to obtain dorsal and a lateral side view with a periodicity of 120 ms. A typical image sequence is depicted in Figure 4, and supplementary videos are available (top view: Video S2, side view: Video S3). Because of the high temporal resolution, the spatial resolution is limited. Nevertheless the body axis can easily be determined, and the limbs are visible in many of the images. Body undulation in the horizontal plane is clearly evident, but there is no bending of the body in the vertical plane. Along with the undulatory movement, the limbs are employed reciprocating in a swinging fashion throughout the observation interval in a similar fashion to the pattern observed during burying. This is evident even when the sandfish is rather deep underneath the sand surface. This indicates that propulsion is, at least in part, generated by the limbs and feet.

**Figure 5:**
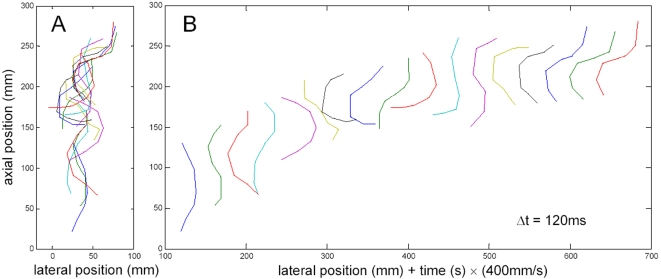
Time series of the behaviour of the longitudinal axis of the sandfish. This series was obtained from the NMR-images shown in Figure 4. The central body axis was measured and depicted showing the principal body shape. (A) Time dependent position and contortion of the length axis of the sandfish from snout to tail. Clearly after submersion in the sand there is always a transverse relative movement between body and sand, i.e. the sandfish body does not glide through a hole made by the snout but shears the sand as it moves. (B) In order to clearly show the sinusoidal shape of the body length axis during the movement, the reconstruction of the length axis was laterally displaced for each time point allowing the determination of amplitude and frequency of the oscillation underlying the sandfish locomotion. Following the lateral displacement of the head, the tail and the centre of the body over time shows an almost identical sinusoidal motion. Clearly similar amplitudes and frequencies of the lateral movement can be observed for all parts of the body.

In order to quantify the animal's movement in the sand, the longitudinal body axis was documented throughout the NMR-image sequences. The result for the image sequence shown in Figure 4 is depicted in Figure 5. Snapshots of the body axis are depicted in different colours for the individual time points (Figure 5A) which were spaced by 120 ms. It is evident that the movement is not a snake-like undulatory pathway through the sand along a constant longitudinal trajectory, but instead, involved significant transverse movements in the sand. To depict the individual snapshots of the body axis, the graphs in Figure 5A were stretched laterally in a time dependent manner by adding a time dependent lateral shift. The result is shown in Figure 5B. Here the shape of the body axis during the movement is clearly seen. The frequency of the meandering movement was again about 3 Hz.

The sandfish depicted in Figures 4 and 5 exhibited a moderate locomotion in longitudinal direction with an initial velocity of about 100 mm/s. In our experiments the velocity of the sandfish in the sand of a depth up to 150 mm ranged up to 300 mm/s.

**Figure 6:**
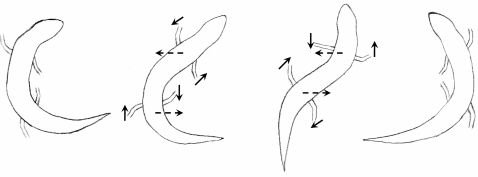
Schematic drawing of the sandfish's movement in the sand for one half-cycle. Dashed arrows indicate the local movement of the body axis while solid arrows indicate the movement of the limbs. The body axis and the limb direction were extracted from figure 4 and 5 respectively comprising NMR-image 7 to 10 in these figures. The limb direction is indicated however, the feet and the exact attitudes of the feet and toes were not drawn, because it is impossible to determine these data from the NMR-images due to the limited resolution.

In order to analyse the movement of distinct parts of the body, the lateral amplitude of the movement of the tip of the head, the mid-part of the body axis and of the tip of the tail were determined. The positions were phase matched. It is clearly evident that the undulations are approximately identical for all parts of the body yielding travelling waves. This can be seen in a schematic drawing of one half-cycle of the sandfish's movement depicted in Figure 6. The drawing is based on the time series shown in Figure 4. While the NMR time series clearly allowed determination of the body axis and the principal movement of the limbs, the exact movement of the feet could not be determined due to the limited spatial resolution.

**Figure 7:**
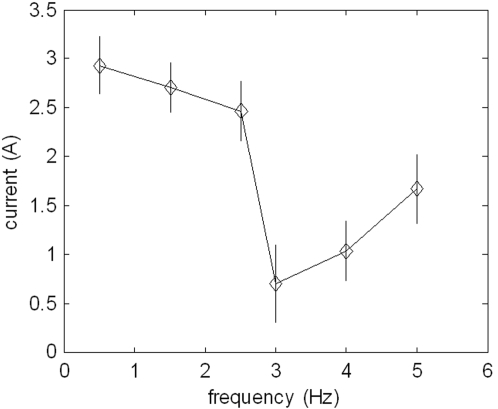
Frequency dependence of the force necessary to move a round body cyclical through sand. A round piece of aluminium was moved using of a home-built apparatus (see supplementary material S5) 100 mm underneath the sand surface in a sinusoidal path with an amplitude of 60 mm. Because the current for the linear motor was controlled to perform the desired movement, the current consumption was a direct measure for the force the motor has to overcome. The minimum of the required current can be seen at about 3 Hz.

As will be discussed below, one can estimate that at certain oscillation frequencies granular media like sand could behave more like a fluid than like a solid body. In order to test whether the undulations at the observed frequency result in a so called decompaction (see below), we measured the power consumption which is direct proportional to the forces necessary to move an aluminium rod of a diameter similar to that of a sandfish (∅20 mm) at certain frequencies through sand in a depth of 100 mm. The results are depicted in Figure 7. Clearly the necessary force drops at about 3 Hz and then slowly increases. In the absence of sand the force is, of course, much lower and increases slightly with increasing frequency (not shown).

## Discussion

In the present study we demonstrate the mode of movement through sand of the sandfish *S. scincus* by using fast imaging NMR. We found a meandering in plane body motion accompanied by a “trotting” like movement of the limbs. No torsion of the body, especially of the head with its spatula-shaped snout was observed. This intuitively logical torsional movement was initially assumed to occur [14]. However, given the tendency of sand to flow, it is clear that the sandfish does not dig tunnels through the loose sand using the snout, but relies upon other methods for progression. It was advocated in accounts published [9], [11], [17], [19], that the meandering movement of the body alone is sufficient to generate the thrust necessary for the movement through the sand. This hypothesis may have been predicted on observations of some lizards (and snakes) swimming in water. Most swimming lizards adpress their limbs against the body during swimming and generate thrust by their body and tail alone [21] (to our knowledge the only exception is another skink, *Tiliqua rugosa* [21]). However, for an undulatory movement to generate thrust, some sort of symmetry-breaking is necessary. This symmetry-breaking can be a modulation of the amplitude of the sinusoidal body movement over the length of the body as observed in water-swimming lizards. When swimming in water, the head remains almost still, whereas the amplitude of the sinusoidal movement increases towards the tail tip [21]. Other possibilities for symmetry-breaking are a modulation of the frequency of a significant change of the body form. In the case of the sandfish swimming in sand, none of these possibilities appear to be adopted. It is evident from the NMR-images that the amplitude and frequency of the sinusoidal lateral movements are almost identical over the entire length of the sandfish. Furthermore, the body does not get broader towards the tail which itself is not laterally compressed. Additionally the limbs are used in the process. We propose that two important effects take place during sand swimming: *i*) the limbs are moved forward in loose sand and backward in compacted (compressed) sand. This leads to a net forward thrust; *ii*) a decompaction of the sand occurs around most of the body of the sandfish.

A complete physical description of the processes in the sand around the “swimming” sandfish is currently under investigation. However, we can estimate the processes taking place in the sand, and do so here by approximating the sandfish problem. Let us first consider the limbs generating thrust. We can only recognise the general characteristics of a trot but cannot distinguish details of the gait pattern [22]. Nevertheless, it can be clearly seen from the time series related the burying, as well as from the NMR-images, that the forward-movement of a limb occurs during bending of the body to the contralateral side, while the backward movement is performed during bending to the ipsilateral (collateral) side (see Figure 6). In the latter case there is pressure from the body applied onto the sand surrounding the limb. Thus the pore-number is reduced leading to an increase in the shear strength of the sand. Conversely, during forward movement of the limb, which is during bending of the body towards the contralateral side, sand flow is induced in the vicinity of the limb leading to a reduction of the shear strength. It can be estimated that a change from theoretically most dense packing (pore-number for quartz sand: *e*∼0.54) to loose packing (pore number *e*∼1) alone will result in a decrease of the shear strength *τ* up to a value of 45%, as *e*·*τ* is approximately constant in dry quartz sand [9], [23]. Thus the backward drag which the limbs generate during forward movement is considerably reduced in comparison to the forward drag during backward movement of the limbs. This effect might be amplified due to coordinated spreading of the toes during retraction. This would lead to a net forward thrust. Here, we should like to add that a detailed analysis of the feet and toe movements would go far beyond the goals of the present study. Although it would be interesting to resolve the exact action of feet and toes, their influence on the decompaction of the sand seems to be only marginal.

**Figure 8:**
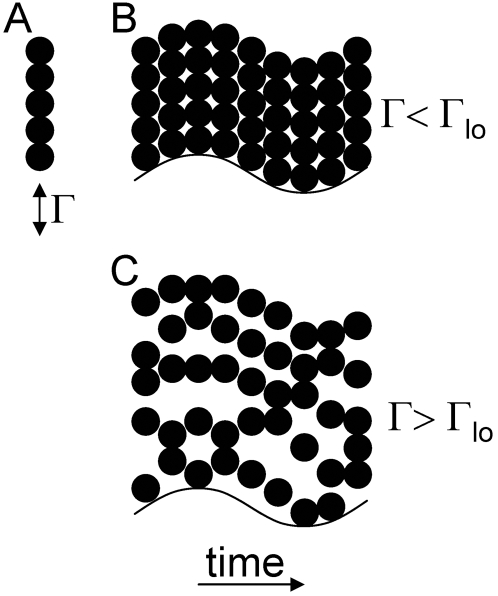
Behaviour of a single column of particles when oscillated vertically. When a single column of particles of a granular medium (A) is oscillated vertically, the particles experience a time-dependent acceleration Γ. If this acceleration is below the lift-off acceleration loΓ (B), the particles stay in contact and the whole column (pile) will stay together and perform the movement: the pile will therefore behave like a solid body. If the acceleration is larger (C), the particles will separate and stay mostly separated due to collisions. The pile will behave more like a fluid.

Additionally, the undulatory movement may lead to a decompaction of the sand surrounding the body of the sandfish. This can be roughly approximated according to the Janssen model [9], in which a sinusoidal vertical movement of a pile of granular medium is assumed. Due to the lateral oscillations of the sandfish, besides particle flows around the body, a vertical acceleration of the sand particles is induced. This is facilitated by the spatula-shaped snout, the streamline body shape with a flattened pentagonal cross section, and the lizard's integument (which exhibits an extremely low friction coefficient for quartz sand [10], [14]). If the vertical oscillation is slow, the sand particles will simply stay in contact and perform the oscillation as a compact pile. If the oscillation is fast, *i.e.* the accelerations the particles experience is above a critical acceleration (the ‘lift-off’ acceleration), the particles will separate and stay separated due to collisions (Figure 8). According to the Janssen model, this phenomenon is called decompaction of the granular material (the sand). This takes place if the acceleration, *Γ*, is beyond the lift-off acceleration, which was found to be *Γ_lo_*≈1.9, according to the calculations, presented in the methods section. This lift-off acceleration is reached at an oscillation frequency of approximately 5 Hz vertical (2.5 Hz oscillation in the horizontal plane). This is clearly below the frequency of the sandfish's meandering motion in the horizontal plane (3 Hz in plane and 6 Hz vertical oscillation respectively). Thus, we may expect a local decompaction of the sand which results in the sand locally behaving more like a fluid than a solid body [23]. The fact that the acceleration is not greatly beyond the lift-off acceleration, but close to it, is not surprising: it would be energetically useless and exhausting for the sandfish to spend a large of effort to further accelerate the sand without having the benefit of a further significant drag-reduction.

Performing force measurements in loose sand clearly showed that at a frequency of 3 Hz, a horizontal sinusoidal movement of a cylindrical probe mimicking the geometry of the sandfish movement required the least force. This experimental result is in excellent agreement with the prediction according to the Janssen model.

Although the above calculation is a rough estimation based on very simplifying assumptions and neglecting some geometrical restrictions, it is instructive to assume that the undulatory movement of the sandfish results in a local decompaction of the sand. This allows the sandfish to “swim” in this fluid-like granular medium by propelling itself forward by using the limbs, which face low drag during forward movement and high drag during backward movement due to the bending of the body. The drag induced by movement through the decompacted sand is orders of magnitude lower than it would be in compact sand [23].

In currently-ongoing work we are attempting to develop a better model for the physical phenomena in the sand around a meandering sandfish based on the NMR-data obtained so far. This might prove useful in two respects. Firstly, we may again a better understanding of the sandfish and other sand-living animals. Secondly, we may be able to learn more about the physics of granular media and some of the means available to effectively decompact these. This might be useful in the engineering sciences in the near future for applications to industrial processing, conveyor technique and handling of grain, sand, powders and the like.

## Materials and Methods

### Photography

Photographic images of the sandfish were taken using a Canon EOS 350D (Canon inc., Tokyo, Japan) with the original telephoto lens (Canon EF-S 18–55 mm, 1∶3.5–5.6) attached. The auto exposure setting was used without flash.

### 3D-Laserscanning

The digital 3D model of the sandfish was acquired using the following process. Firstly the specimen was scanned from 26 different vantage points using a Minolta VI-900 non-contact laser range digitizer. The scanner casts a moving plane of laser light onto the object to be digitized and captures, for each position of the plane, an image of the laser projection using a CCD camera. The object's shape is then derived from the sequence of observed contours (see [24] for more details on optical triangulation). The scanner's output is a depth-image related to each vantage point, which are then easily transformed into a surface patch. Since the positions and orientations of the resulting surface patches are not aligned after scanning, the patches have to be transformed into a common coordinate frame. This was done using a custom implementation of the Iterative Closest Points (ICP) algorithm [25]. Finally the individual surface patches were merged to a single watertight triangle mesh by applying the surface reconstruction method [26].

### NMR - imaging

The movement of the sandfish was measured in a MRI-compatible setup with a time resolution of 120 ms and a spatial resolution of 1.56 mm×1.17 mm. The experimental setup was optimized for a sufficient signal-to-noise ratio, while allowing the sandfish to move through the sand. Rapid 3D imaging was done using a newly developed MRI method, as described below. Three individuals were investigated.

#### Experimental Setup

The imaging experiments were performed using a 1.5 T whole-body scanner (Magnetom VISION; Siemens Medical Systems, Erlangen, Germany) with a peak gradient amplitude of 25 mT/m and a slew rate of 83 T/m/second. For signal reception, a head coil was used in all measurements. The sensitive area of this coil has a length of approximately 30 cm. Signal transmission was done using the built in body coil. Since the sandfishes, and especially the sand, did not load the body coil sufficiently, additionally four bottles, each filled with 1.5 litres of water containing nickel sulphide (NiSO_4_), were also placed symmetrically around the head coil. T1 and T2 relaxation time was due to high concentration of nickel sulphide less than 0.1 ms, meaning, that equilibrium magnetization is reached directly after excitation. No MRI-signal were generated from this additional load since typical echo times for imaging methods and especially the described method is typical beyond 1 ms. The coil was filled with a cylindrical box, 40 cm long, and 22 cm in diameter. The size was chosen to fill the imaging volume of the receiver coil. The lower 60% of the box was filled with “ExoTerra Desert Sand - White” with a grain size of 0.1–1 mm. In the head direction there was an entrance with an attached platform directly above the sand surface. The sandfish was set on the platform. In all cases the animals moved directly into the sand surface, quickly burying themselves. Typical MRI scanners, such as the VISION, require a setup procedure for each measurement. Machine settings for the following imaging experiment rely on these parameters. During setup an object generating MR-signal is required in an isocentric axial plane. Therefore during the setup procedure a 50 ml water-filled centrifuge tube (Thermo Fisher Scientific, Roskilde, Denmark) was placed in the magnet isocenter. This tube was removed before the sandfish measurement was started.

The three dimensional and time resolved detection of sandfish movement was started when the sandfish was placed on the platform. The entire burying process and the movement within the sand was imaged until the sandfish settled in its final position.

#### Imaging protocol

The newly developed MRI sequence acquires two orthogonal projections of the entire volume with a high resolution. These projections show only the signal from the sandfish, because the sand does not contribute to the MRI signal. The coronally oriented projection delivers an image showing the sandfish as seen in dorsal view, which reveals the shape of the body in the horizontal plane and also the position of the feet during movement (Figure 4). The transverse projection depicts the body shape as seen from the side. From this, one can determine the depth of the sandfish within the sand filled container. Since the sandfish is approximately a concave object, all necessary information concerning spatial position, movement and foot position can be derived from the acquired image series. To achieve a high temporal resolution, which is necessary for monitoring sandfish movement, image folding in phase encoding direction was beneficially used. In typical MRI measurements it appears as an unwanted artefact, but in this case it allows to image with a high spatial resolution with the need to cover only a small field of view (FOV) resulting a fast image acquisition. All objects exceeding, or in this case moving out of, the field of view in the phase-encoding direction, will appear with same intensity on the opposite side. If the FOV is adjusted to the dimension of the sandfish instead of the whole setup including the sand, imaging speed can be increased, and always delivers a complete image of the reptile. This conserves the possibility of monitoring arbitrary 3D movement of the sandfish with high resolution. Read encoding direction was chosen for both projections in bore direction of the scanner. Therefore the FOV was chosen to 300 mm in read direction to cover the entire sand bowl and 37.5 mm in phase encoding direction to guarantee that no body parts of the sandfish fold on each other.

#### Imaging sequence

Simultaneous imaging of both orthogonal projections was done with a modified FLASH sequence [27] with double echo readout. In order to obtain projection of the entire setup, no slice selection gradient was used. Read encoding gradient was chosen for both echoes in axial direction. The first echo was phase encoded in coronal direction. After the first echo acquisition, all gradients were refocused. The second echo was phase encoded in transversal direction. A schematic of the pulse sequence can be seen in figure 4.

Imaging parameters were optimized to a FOV of 300 mm×37.5 mm and a matrix size of 24×256 pixels, resulting in a spatial resolution of 1.56 mm×1.17 mm. Duration for both projection images was 120 ms, resulting in an image rate of 8 times two orthogonal projections per second. The repetition time T_R_ of the FLASH sequence was 5.0 ms. The echo time T_E_ of the first echo was 1.2 ms and of the second echo 3.9 ms. The nominal flip angle α was optimized for imaging of the sandfish experimentally to 8°. Since the flip angle calculation is based on the scanner setup procedure done with a 50 ml water tube, it is assumed, that the real flip angle in sandfish can differ from this value. In total, 128 consecutive sets of two orthogonal projections were taken, covering the first 16 seconds of sandfish movement.

### Mathematical model

For description of the granular sand surrounding the sandfish, the Janssen model was used [23]. This model describes the decompaction of granular media, *i.e.* a transition from a solid like to a fluid like behaviour in the case of a vertical acceleration of the medium. In our case, each point on the axis of the sandfish moves laterally as
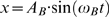
With *A_B_* standing for the amplitude of the meandering motion of the sandfish, *ω_B_* is the angular frequency of this movement and *t* denotes time. The meandering movement of the lizard in the horizontal plane will induce a sinusoidal vertical movement of the surrounding sand. The vertical acceleration of the sand due to the body moving in the described manner would be
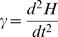
with *H* being the body height at a certain position at a certain time. In our case when approximating the body cross section by a circle of diameter *A*, we can do a Fourier-series approximation of the above formula. Using only the first term of the series, *i.e.* the basic frequency *ω*, we obtain a vertically acceleration of *γ*≈*Aω^2^* with *A* as the bodies diameter, *i.e.* the maximal body height and *ω* stands for the angular frequency of the up- and down-oscillation. As during a single cycle of the sandfish in the horizontal plane the sand is moved up and down twice, *ω* = *2ω_B_*. Thus the frequency of vertical acceleration results from the oscillation frequency of the body, which is about 3 Hz, to be approximately 6 Hz. In our case the amplitude, *i.e.* the body's diameter is about 20 mm. For convenience we introduce the dimensionless acceleration *Γ* = *γ/g* with *g* being the gravitational acceleration.

According to the theory on the Janssen model a so called decompaction of the granular material, *i.e.* the sand takes place, when the acceleration *Γ* is beyond the lift-off acceleration which was found to be
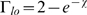
with χ being the decompaction parameter which is defined as
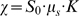

[23]. Here *S*_0_ describes the aspect ratio of the geometry, *μ_s_* is the static granular friction coefficient of the medium and *K* is a parameter comprising the stacking of the granular medium. For a compact triangular stack (dense globular packing) the parameter *K* = 0.58 and the static friction coefficient for sand was found to be about 0.7 [23]. The aspect ratio *S*_0_ describes the ratio of the height of the sand pile (*i.e.* the immersion depth of the sandfish) times the perimeter of the pile divided by the cross section of the pile. In our case, if we assume the pile of height 100 mm to be rectangular over the moving sandfish which has a length of 120 mm and is oscillating about 60 mm laterally, we obtain a lift-off acceleration of *Γ_lo_*≈1.9. Assuming the sandfish diameter 20 mm, the lift-off acceleration will be reached at a frequency for the vertical oscillation of the sand pile of 5 Hz which corresponds to an oscillation of the sandfish in the horizontal plane with 2.5 Hz.

### Drag measurements in sand

For determination of the force necessary to move a non flexible body of the diameter of a sandfish through loose sand sinusoidally, a 150 mm long aluminium rod with diameter 20 mm was mounted at the end by a tin construction to a linear motor (LinMot PS 01-23x80, Linmot Coorp. Spreitenbach, CH) which was controlled by a E 100 controler (LinMot). The controller was commanded by a PC using the manufacturer's controller software for the E 100. This allowed performing sinusoidal motions of the aluminium rod in bowl of sand at a depth of 100 mm with an amplitude of about 60 mm and defined frequencies ranging from 0.5 Hz to 6 Hz. The force necessary to perform the programmed movement could be directly recorded via the controller by measuring the current consumption over time. This current consumption is, according to the manufacturer, direct proportional to the axial force of the linear motor. The tin construction hardly influenced the results as only the thickness (1 mm) of the tin faced the moving direction. The measuring device is depicted in the supplementary material (Figure S2). The measurements were carried out using the ExoTerra desert sand also used for the observations of the sandfish.

## Supporting Information

Figure S1 3D-polygonal surface model of a sandfish obtained by 3D-laser scanning. Best seen with quich3D viewer when saved as OBJ-file.
(0.40 MB TXT)

Figure S2 Photograph of the used force measuring apparatus
(0.39 MB JPG)

Video S1 Real time video of the burying behaviour of an adult sandfish observed from above.
(1.51 MB AVI)

Video S2 NMR-imaging sequence of a sand-swimming skink in top view.
(1.35 MB MOV)

Video S3 NMR-imaging sequence of a sand-swimming skink in side view.
(1.35 MB MOV)
