# Supplementary figures and images for: Investigating the Locomotion of the Sandfish in Desert Sand Using NMR-Imaging
Source: PLoS One. 2008 Oct 1;3(10):e3309. doi: 10.1371/journal.pone.0003309 (PMC2561000; doi:10.1371/journal.pone.0003309)

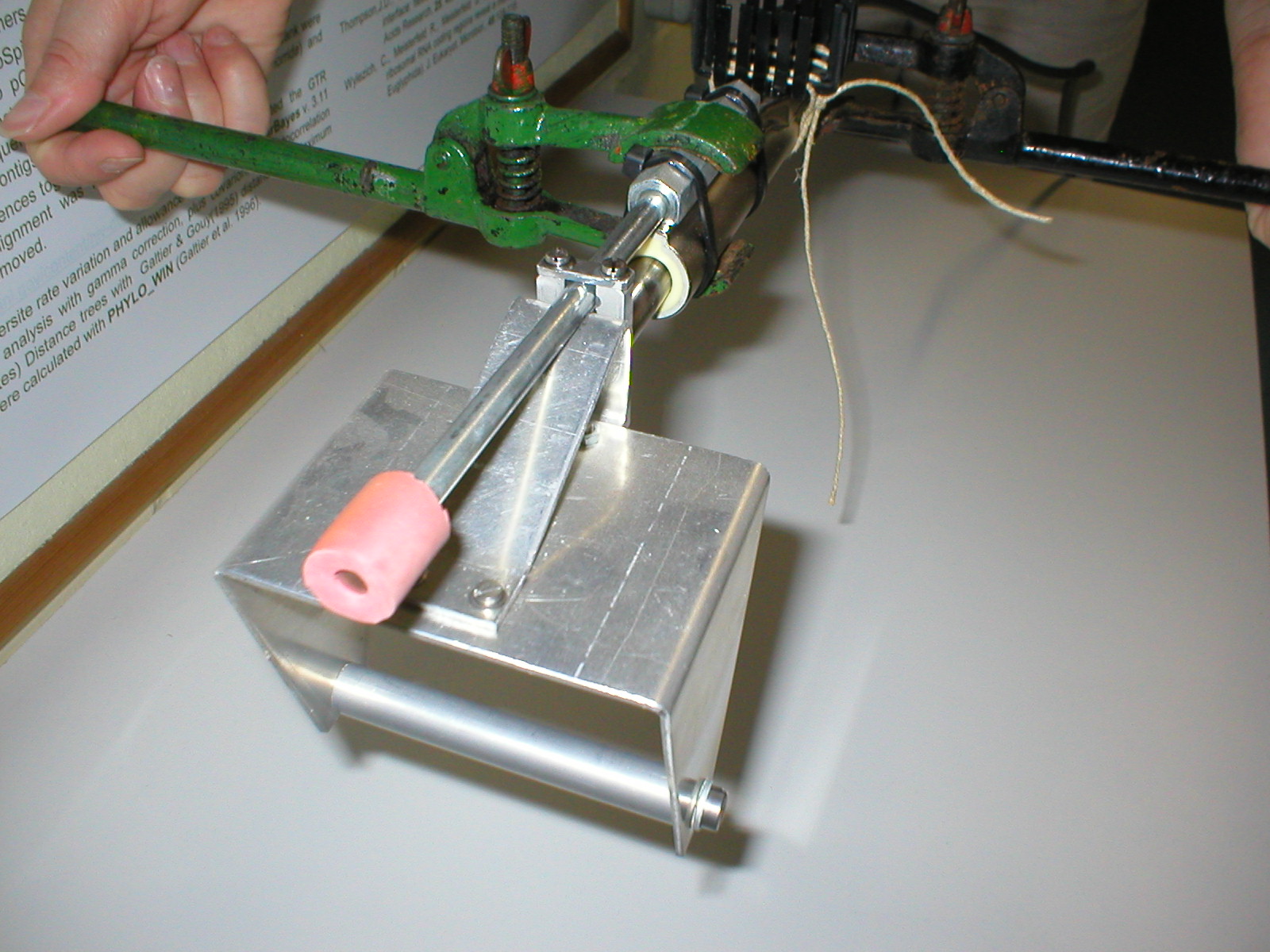

Supplement: Figure S2 — Photograph of the used force measuring apparatus (0.39 MB JPG) [file pone.0003309.s002.jpg]
